# COVID-19 pandemic phases and female precocious puberty: The experience of the past 4 years (2019 through 2022) in an Italian tertiary center

**DOI:** 10.3389/fendo.2023.1132769

**Published:** 2023-02-28

**Authors:** Laura Chioma, Mariangela Chiarito, Giorgia Bottaro, Laura Paone, Tommaso Todisco, Carla Bizzarri, Marco Cappa

**Affiliations:** Endocrinology Unit, University Hospital Pediatric Department, Bambino Gesù Children’s Hospital, Istituto di Ricovero e Cura a Carattere Scientifico (IRCCS), Rome, Italy

**Keywords:** precocious puberty, lockdown, COVID-19, pandemic, girls

## Abstract

**Objective:**

Since the outbreak of COVID-19 pandemic, several centers of pediatric endocrinology worldwide have observed a significant increase in the number of girls presenting with precocious or early puberty. We aimed to compare the incidence rates of female precocious puberty before and during the different phases of COVID-19 pandemic.

**Methods:**

We have retrospectively analyzed all the consultations recorded in the outpatient clinic database of the Endocrinology Unit of Bambino Gesù Children’s Hospital, Rome, Italy, from the lockdown start in March 2020 up to September 2020, in comparison with the consultations recorded in the same months of 2019, 2021 and 2022. Age, height, weight, body mass index, Tanner’s pubertal stage and bone age at presentation, birth weight, ethnicity, family history of central precocious puberty (CPP), maternal age at menarche, history of adoption were retrieved from clinical records. Serum levels of follicle-stimulating hormone (FSH), luteinizing hormone (LH) both at baseline and after gonadotropin-releasing hormone (GnRH) stimulation, and basal estradiol levels were collected.

**Results:**

In 2019, 78 girls with suspected precocious puberty were referred for endocrinological consultation, compared to 202 girls in 2020, 158 girls in 2021 and 112 girls in 2022. A significant increase in the proportion of girls diagnosed with rapidly progressive CPP was observed in 2020, compared to 2019 (86/202 *vs.* 18/78, p<0.01). In the following periods of 2021 and 2022, a gradual decrease in the number of cases of progressive CPP was evident, so much that the number of cases was not significantly different from that observed in 2019 (56/158 in 2021 and 35/112 in 2022, p=0.054 and p=0.216 respectively, compared to 2019).

**Conclusions:**

Our research suggests that drastic lifestyle changes, such as those imposed by COVID-19 lockdown, and the consequent stress may affect the regulation of pubertal timing. The remarkable increase in CPP cases observed during the 2020 first pandemic wave seems to be reduced in 2021 and 2022, concurrently with the progressive resumption of daily activities. These data seem to support the hypothesis of a direct relationship between profound life-style changes related to the pandemic and the rise in precocious puberty cases.

## 1 Introduction

Puberty is the crucial transition process between childhood and adulthood, leading to full reproductive capacity (1). Female central precocious puberty (CPP) is defined as the onset of breast development before the age of eight years, due to the activation of the hypothalamic-pituitary-ovarian (HPO) axis (2). Puberty is a complex phenomenon, and factors modulating timing and/or tempo of puberty are not fully understood. It has been assumed that genetic, epigenetic and environmental factors, such as energy imbalance, exposure to endocrine disruptors or stressful events may trigger an earlier pubertal development (3, 4).

In the last century a trend toward earlier puberty was already observed (5–7). This phenomenon, known as “secular trend of puberty”, has described a progressive reduction in the age at menarche, dropping from 17 years in the early-1800s to 13 years by the mid-1900s, with a further minor decline through the last three decades (5).

Recently, several centers of pediatric endocrinology worldwide, including ours, have observed a further significant increase in the number of girls presenting with precocious or early puberty since mid-2020 (8–21). During this period, corresponding to the first wave of COVID-19 pandemic, the Italian government imposed a strict lockdown across the country, in order to reduce the transmission rate and to avoid hospital bed saturation. Consequently, profound changes in everyday life occurred, such as school closures and the restriction of outdoor and team sports activities. Families were forced to stay at home, except for emergency reasons, with more opportunities for hypercaloric food consumption and overnutrition and the worsening of sedentary lifestyle. There was a significant rise of e-learning, extremely uncommon in primary schools before the pandemic. All these changes led to a larger daily use of electronic devices among children.

Given the growing worldwide evidence of an increase in female precocious puberty since the outbreak of COVID-19 pandemic, we aimed to investigate the evolution of this phenomenon before and during the different phases of the pandemic, from 2019 to 2022.

## 2 Materials and methods

### 2.1 Subjects

We retrospectively analyzed all the consultations for suspected precocious or early puberty recorded in the outpatient clinic database of the Endocrinology Unit of Bambino Gesù Children’s Hospital, Rome, Italy from lockdown start in March 2020 to September 2020, in comparison with the consultations recorded in the same period of 2019, 2021 and 2022.

Consultations for premature thelarche in girls younger than 3 years were excluded. All subjects with suspected precocious puberty were observed for up to three months in order to reach the final diagnosis.

For each year, the subjects were further divided into subgroups based on the final diagnosis: transient thelarche (TT), non-progressive precocious puberty (NPP), CPP, or early puberty (EP). Subjects presenting with thelarche that disappeared during the 3-month observation period were assigned to the TT group. EP was defined as pubertal signs first appearing between 8 and 9 years, these girls were not further investigated.

The Institutional Review Board of ‘Bambino Gesù Children’s Hospital approved the study protocol.

### 2.2 Anthropometric data and medical history

Age, height (H), weight (W), body mass index (BMI), pubertal stage and bone age (BA) at presentation, birth weight, ethnicity, CPP family history, maternal age at menarche, history of adoption were retrieved from clinical records. H (cm) and W (kg) were also expressed as age and sex specific standard deviation score (SDS) according to the standard growth charts for the Italian population (22). Body mass index (BMI) was calculated as the ratio between W and H^2^ and expressed as SDS. Birth weight was expressed also as SDS according to the Italian Neonatal Anthropometric Charts (23). Tanner’s method was used to assess pubertal stages (24). Questionnaires concerning physical activity, screen time and eating habits at the onset of pubertal signs were administered to all groups.

### 2.3 Laboratory measurements

Serum levels of follicle-stimulating hormone (FSH), luteinizing hormone (LH) both at baseline and after gonadotropin-releasing hormone (GnRH) stimulation, and basal estradiol levels were collected, when available, among all subgroups except EP. GnRH stimulation test was performed by the i.v. administration of GnRH (Lutrelef; Ferring) at a dosage of 100 µg, with FSH and LH measurement at baseline and 30, 60 and 90 minutes after the injection. A basal LH level above 0.2 IU/l and/or a LH peak after GnRH infusion above 5 IU/l were considered diagnostic for CPP (2, 25). In the absence of one or both these criteria, subjects with slow pubertal progression were assigned to the NPP group.

### 2.4 Imaging

All subjects underwent pelvic ultrasound to assess uterine and ovarian characteristics. A uterine longitudinal diameter above 36 mm and the presence of the endometrium echo-pattern were considered signs of estrogenic stimulation, suggestive of precocious puberty.

An X-ray of the left hand and wrist was performed in all subjects to assess BA, according to the Greulich & Pyle method (26). Bone age advancement (years) was assessed as the difference between BA and chronological age.

Most subjects diagnosed with CPP (152 girls, 78%) underwent a magnetic resonance of the hypothalamus-pituitary area to rule out intracranial pathologies.

### 2.5 Statistical analysis

Data were expressed as mean ± SD when normally distributed and as median (interquartile range or IQR) for parameters with non-normal distribution, unless otherwise specified. Categorical variables were reported as number and percentage. The observed subjects were divided into four groups according to the year of evaluation (2019, 2020, 2021 or 2022). Each group was further divided in four subgroups according to the final diagnosis (TT, NPP, CPP or EP). Categorical variables were compared using chi-square (χ²) test. ANOVA was applied to compare variables with normal distribution between more than two groups, while Kruskal–Wallis test was applied for variable with non-normal distribution.

Statistical analysis was performed with the statistical package SPSS v23 for Windows (SPSS Inc, Chicago, IL, USA) and a probability value of p < 0.05 was considered statistically significant.

## 3 Results

The sharpest increase of consultations was observed in 2020, with 208 subjects referred for suspected precocious or early puberty among a total number of 747 consultations in the period March-September 2020 (27.8%), in comparison with 85 subjects/1260 consultations in the same period of 2019 (6.7%). In 2021 there was still an increase in consultations for suspected precocious puberty, even if less pronounced than in 2020, with 166 subjects/1190 consultations (13.3%). A further reduction of consultations was observed in 2022, with 120 subjects/1380 consultations (8.7%).

Given the similarity in the number of boys observed throughout the years (7 subjects in 2019, 6 subjects in 2020, 8 subjects in 2021 and 8 subjects in 2022), we decided to further analyze only the female population of each considered period. Thirty-one girls were excluded because they were lost at follow-up after the first observation.

**Figure 1:**
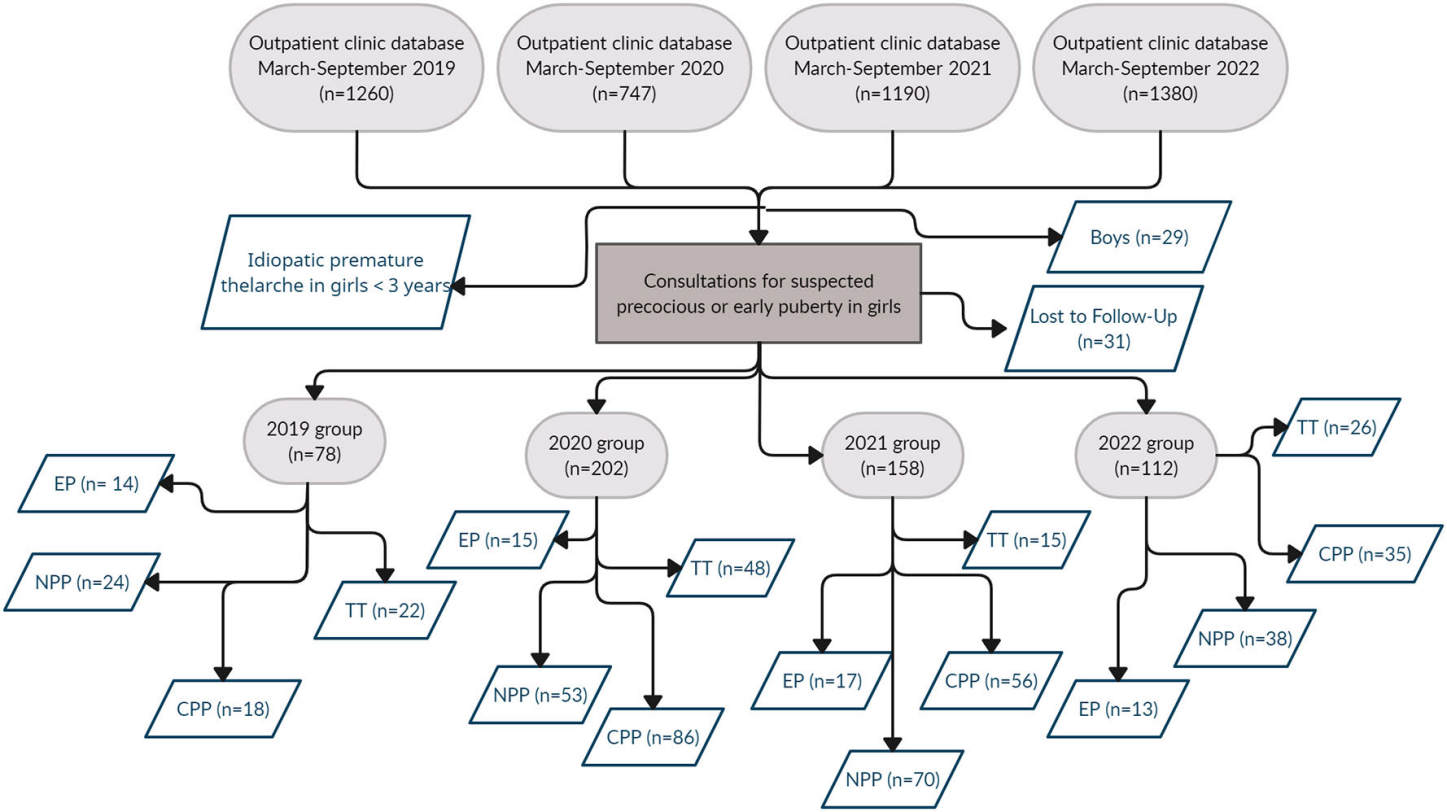
Flowchart summarizing the study design.

The study population consisted of 550 girls, divided as follows: 78 girls evaluated in 2019, 202 girls in 2020, 158 girls in 2021 and 112 girls in 2022. **Figure 1** summarizes the design of the study and the results of data collection.

The number of consultations for suspected precocious or early puberty in girls was confirmed significantly higher in 2020 than in 2019 (202/747 equivalent to 27% in 2020 *vs.* 78/1260 equivalent to 6.2% in 2019, p<0.01). In 2020, the most evident increase in consultations was observed during the months following the lockdown (139/202 between June and September, equivalent to 72.8% *vs.* 63/202 between March and May, equivalent to 27.2%). In 2021 an initial downward trend was observed (158/1190, equivalent to 13.3%, p<0.01 *vs.* 2020), that became even further evident in 2022 (112/1380 equivalent to 8.1%, p<0.01 *vs.* 2020). This progressive downward trend led to a number of consultations in 2022 that was not significantly different from the number observed in 2019 (8.1% *vs.* 6.2% respectively, p=0.06) (**Table 1**).

**Table 1:** Consultations for suspected precocious or early puberty recorded between March and September from 2019 to 2022, in comparison with the overall consultations recorded in the same period of the years.

	Visit for suspected precocious puberty between March-September			Total visit March-September
	March-May (%)	June-September (%)	Total (%)	
2019	37 (47.4)	41 (52.6)	78 (6.2)*	1260
2020	63 (27.2)	139 (72.8)	202 (27)*	747
2021	77 (48.7)	81 (51.3)	158 (13.3)*	1190
2022	48 (42.9)	64 (57.1)	112 (8.1)*	1380

*p<0.01 for 2020 vs. 2019, 2021 and 2022.

CPP family history was positive in 28.7% of girls in 2020, in 24.1% in 2021 and in 31.3% in 2022, without significant differences with the 2019 population (35.9%). In total, ten girls had been adopted, 3 of them belonged to the NPP group and 7 to the CPP group.

**Figure 2:**
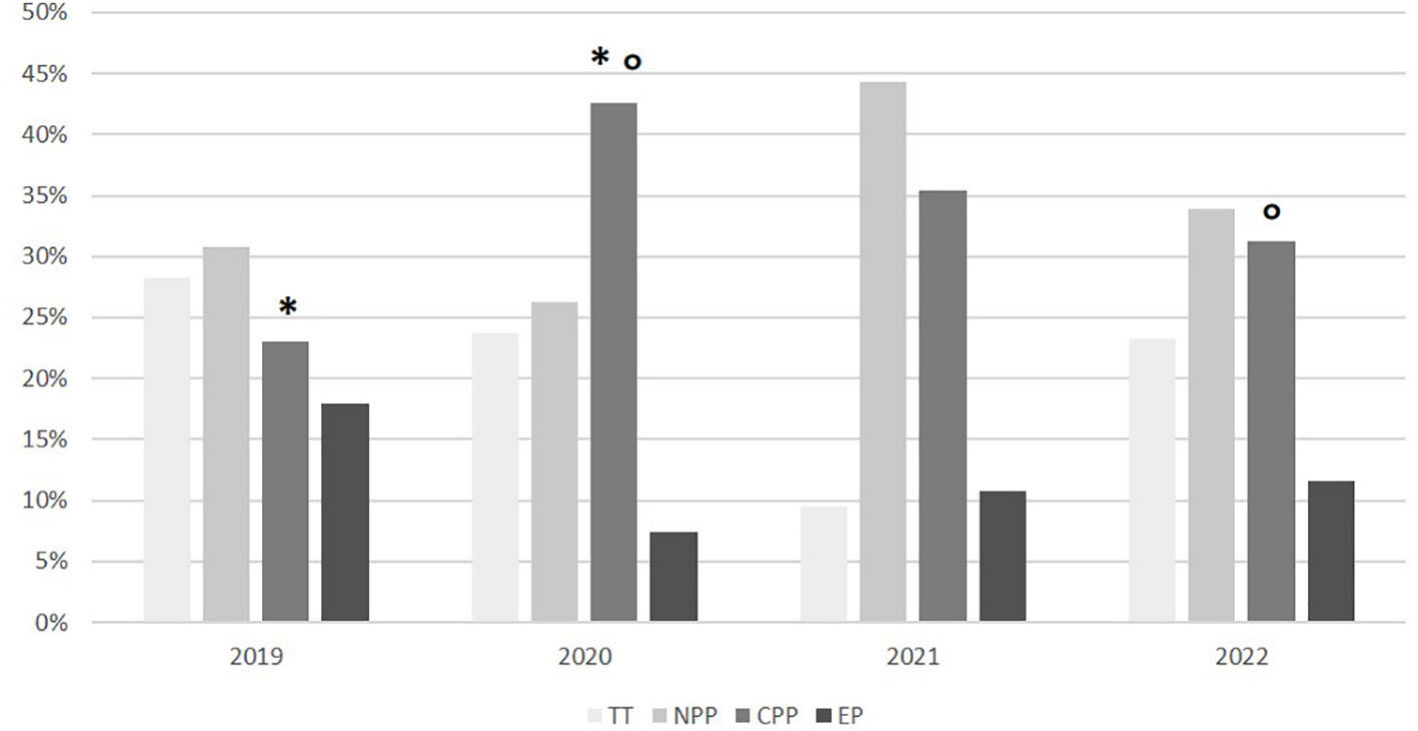
Subgroup distribution according on the final diagnosis in the different years. TT, transient thelarche; NPP, non-progressive precocious puberty; CPP, central precocious puberty; EP, early puberty. *p<0.01; °p<0.05.

The proportion of girls with rapidly progressive CPP was significantly higher in 2020, compared to 2019 (86/202 *vs.* 18/78, equivalent to 42.6% *vs.* 23.1%, p<0.01). In 2021, the number of cases of progressive CPP slightly decreased, compared to 2020 (56/158 *vs.* 86/202, equivalent to 35.4% *vs.* 42.6%, p = 0.17). In 2022, a further significant reduction in the number of cases of progressive CPP was observed compared to 2020 (35/112 *vs.* 86/202, equivalent to 31.3% *vs.* 42.6%, p=0.04). The number of cases observed in 2022 was not statistically different from the number of cases observed in 2019 (35/112 *vs.* 18/78, equivalent to 31.3% *vs.* 23.1%, p=0.22) (**Figure 2**).

**Table 2** shows patients’ characteristics according to the year of observation and final diagnosis.

**Table 2:** Anthropometric parameters of study population according to year of observation and final diagnosis.

		Number (%)	Age (years)	Height SDS	Weight SDS	BMI SDS	BW SDS	BMI SDS – BW SDS
2019	TT	22 (28.2)	6.72 ± 0.81	0.57 ± 1.10	0.62 ± 1.07	0.55 ± 1.03*	-0.05 ± 1.12	0.57 ± 1.49
	NPP	24 (30.8)	7.34 ± 0.67°	0.99 ± 1.10	0.71 ± 1.15	0.42 ± 1.20	0.07 ± 1.05	0.30 ± 1.39
	CPP	18* (23.1)	7.02 ± 0.94	1.15 ± 1.01	0.87 ± 0.82	0.64 ± 0.61	-0.06 ± 0.85	0.65 ± 0.80
	EP	14 (17.9)	8.28 ± 0.39	0.84 ± 0.94	0.45 ± 1.01	0.20 ± 1.09	-0.09 ± 0.82	0.27 ± 1.41
2020	TT	48 (23.8)	6.89 ± 0.98	0.45 ± 0.98	-0.03 ± 0.86	-0.29 ± 0.90*	0.04 ± 1.29	-0.25 ± 1.38
	NPP	53 (26.2)	6.83 ± 0.84°	0.58 ± 1.01	0.50 ± 0.96	0.38 ± 0.96	-0.17 ± 1.08	0.58 ± 1.20
	CPP	86 (42.6)*°	7.05 ± 0.70	0.88 ± 0.94	0.40 ± 1.08	0.01 ± 1.80	-0.15 ± 1.01	0.24 ± 2.02
	EP	15 (7.4)	8.15 ± 0.35	0.52 ± 1.13	0.18 ± 1.10	0.02 ± 1.10	0.09 ± 1.09	-0.20 ± 0.99
2021	TT	15 (9.5)	6.55 ± 0.97	-0.01 ± 1.21	-0.12 ± 1.11	-0.08 ± 1.02	-0.01 ± 1.23	-0.27 ± 1.38
	NPP	70 (44.3)	7.13 ± 0.83	0.62 ± 0.93	0.69 ± 1.08	0.58 ± 1.15	-0.17 ± 0.99	0.77 ± 1.60
	CPP	56 (35.4)	7.27 ± 0.53	0.85 ± 0.99	0.57 ± 0.76	0.37 ± 0.77	-0.25 ± 1.23	0.64 ± 1.34
	EP	17 (10.8)	8.14 ± 0.44	1.00 ± 0.72	0.35 ± 0.69	-0.05 ± 0.84	-0.37 ± 1.12	0.12 ± 1.00
2022	TT	26 (23.2)	6.43 ± 0.94	0.41 ± 0.88	0.13 ± 0.94	-0.02 ± 0.89	-0.12 ± 0.94	0.08 ± 1.17
	NPP	38 (33.9)	7.26 ± 0.60°	0.70 ± 0.84	0.43 ± 0.91	0.22 ± 1.04	-0.05 ± 1.13	0.20 ± 1.22
	CPP	35 (31.3)°	7.30 ± 0.47	1.05 ± 1.17	0.60 ± 1.04	0.28 ± 1.11	-0.09 ± 1.40	0.28 ± 1.38
	EP	13 (11.6)	8.04 ± 0.67	0.66 ± 0.95	0.60 ± 0.88	0.54 ± 0.82	-0.22 ± 1.29	0.61 ± 1.04

CPP, central precocious puberty; EP, early puberty; NPP, non-progressive precocious puberty; TT, transient thelarche; BW, Birth Weight; BMI, Body Mass Index. *p<0.01; °p<0.05.

Parameters are expressed as mean ± SD if not differently indicated.

No significant differences in anthropometric characteristics and laboratory parameters were found comparing the CPP subgroups of the four different years. The exceptions to this finding were a lower basal LH level in 2020 compared to 2022 (0.7 ± 0.98 IU/L in 2020 *vs.* 1.88 ± 1.99 IU/L in 2022, p<0.01) and a less evident BA advancement in 2020 compared to 2021 (1.32 ± 0.92 years in 2020 *vs.* 1.85 ± 1.17 years in 2021, p=0.02) (**Table 3**).

**Table 3:** Anthropometric characteristics and laboratory/instrumental parameter comparing the central precocious puberty (CPP) subgroups in the four different years.

CPP	2019	2020	2021	2022
Number (%)	18 (23.1)*	86 (42.6)*°	56 (35.4)	35 (31.3)°
Age (years)	7.02 ± 0.94	7.05 ± 0.70	7.27 ± 0.53	7.30 ± 0.47
Birth Weight SDS	-0.06 ± 0.85	-0.15 ± 1.01	-0.25 ± 1.23	-0.09 ± 1.40
Height SDS	1.15 ± 1.01	0.88 ± 0.94	0.85 ± 0.99	1.05 ± 1.17
Weight SDS	0.87 ± 0.82	0.40 ± 1.08	0.57 ± 0.76	0.60 ± 1.04
BMI SDS	0.64 ± 0.61	0.01 ± 1.80	0.37 ± 0.77	0.28 ± 1.11
BMI SDS – BW SDS	0.65 ± 0.80	0.24 ± 2.02	0.64 ± 1.34	0.28 ± 1.38
Basal LH (IU/L)	1.00 ± 1.51	0.52 ± 0.98*	1.13 ± 1.22	1.88 ± 1.99*
LH peak (IU/L)	19.49 ± 16.79	17.01 ± 14.02	22.48 ± 17.35	21.28 ± 14.27
17-beta-estradiol (pg/mL)	8.50 ± 10.02	16.54 ± 19.25	16.18 ± 18.26	24.23 ± 20.45
BA - CA	1.69 ± 0.75	1.32 ± 0.92°	1.85 ± 1.17°	1.82 ± 1.06
Uterine longitudinal diameter (mm)	42.04 ± 6.85	38.83 ± 8.02	41.30 ± 9.23	41.88 ± 7.65

Parameters are expressed as mean ± SD if not differently indicated. BMI, body mass index; BW, birth weight; BA, bone age; CA, chronological age. *p<0.01; °p<0.05.

The majority of CPP girls (78%) underwent brain MRI study, none of them showed organic lesions related to CPP.

**Figure 3:**
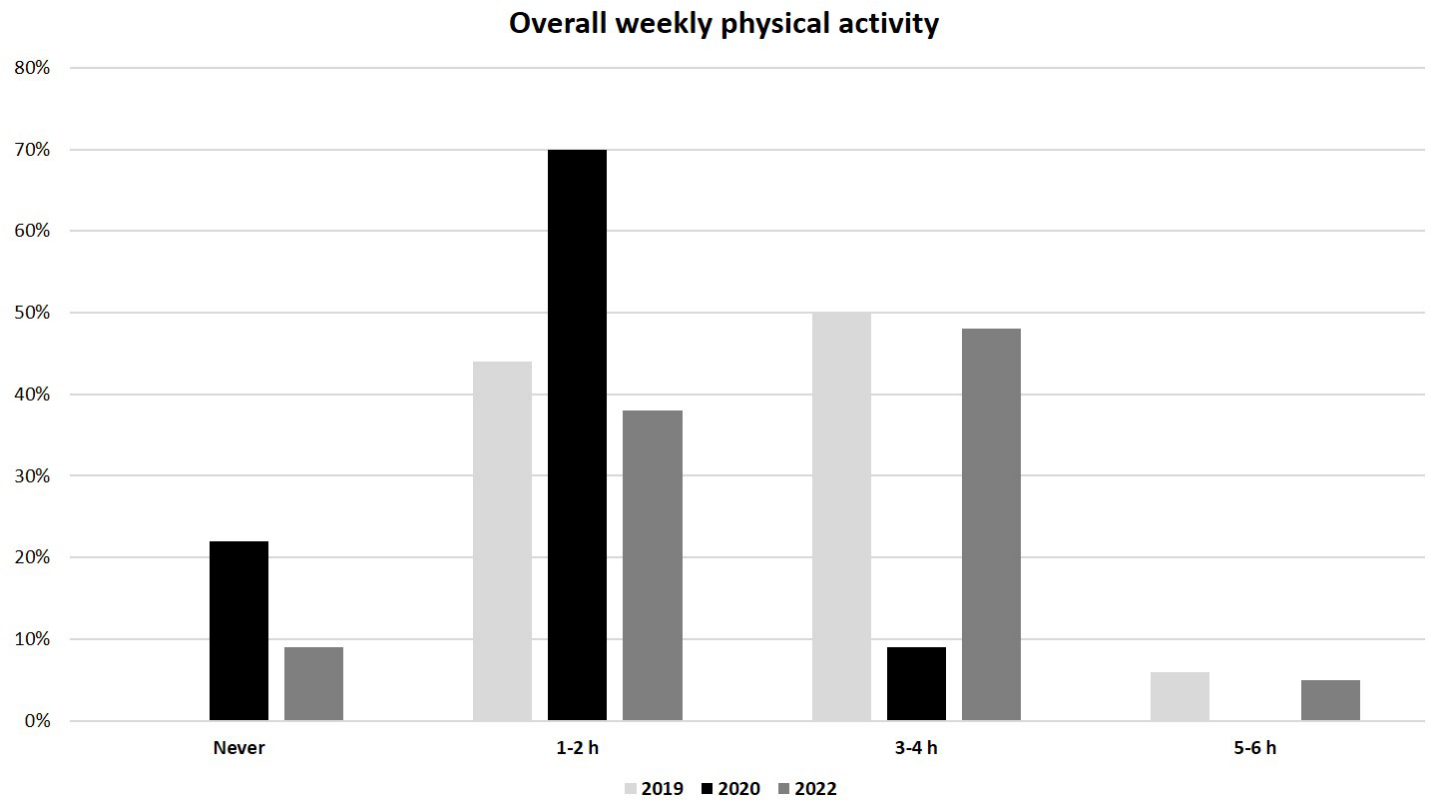
Overall weekly physical activity in 2019, 2020 and 2022 populations.

**Figure 4:**
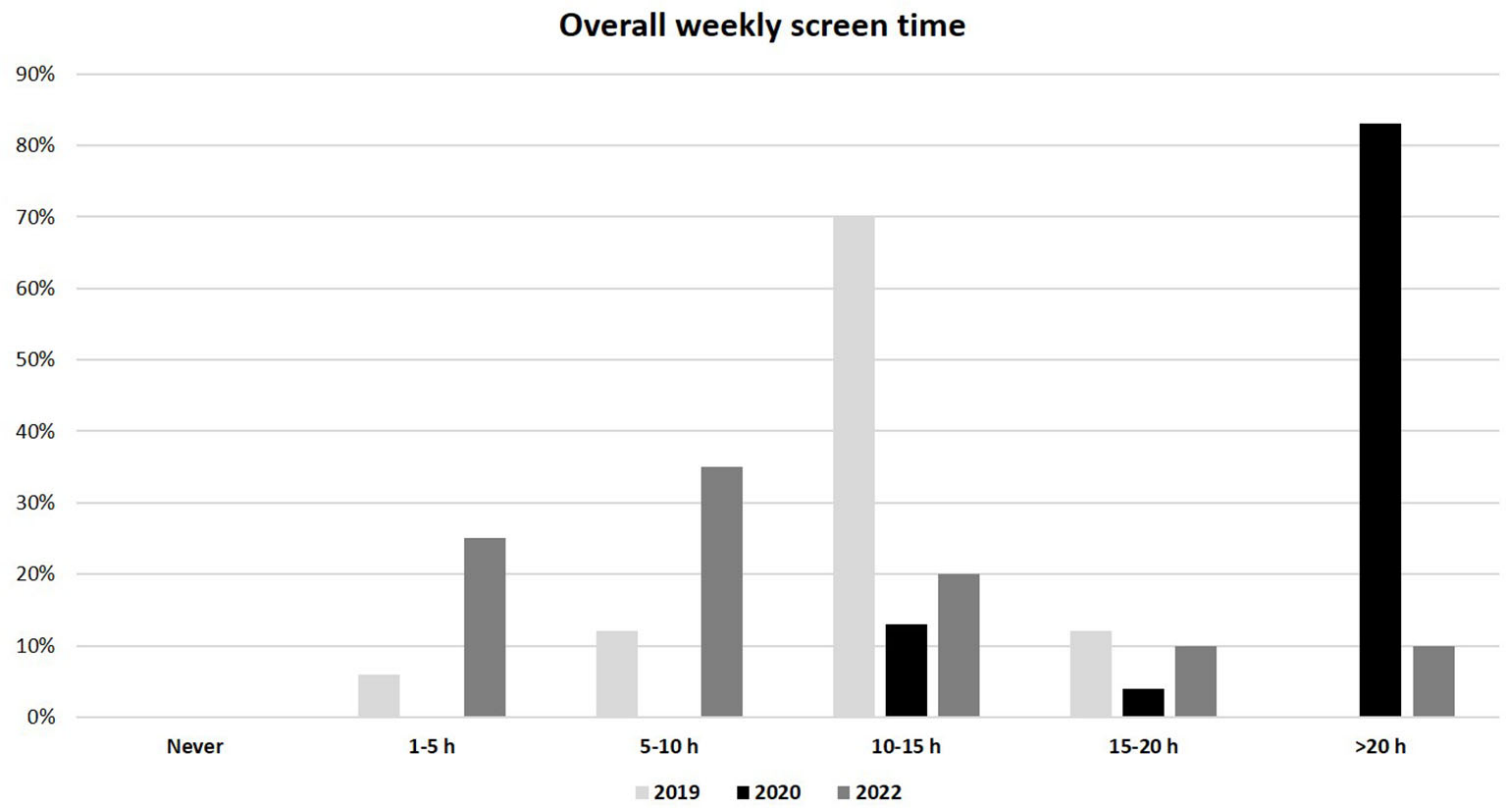
Overall weekly use of electronic devices (as tablet, PC and smartphone) in 2019, 2020 and 2022 populations.

As regards to lifestyle, a significantly lower weekly physical activity was reported in the 2020 group compared to the 2019 and 2022 groups (median 1-2 h/week, IQR (0) in 2020 *vs.* 3-4 h/week, IQR (1-2 h/week to 5-6 h/week) in both 2019 and 2022, p<0.01) (**Figure 3**). In addition, the overall weekly time spent on electronic devices (as tablet, PC or smartphone) was considerably greater in the 2020 group than in 2019 and 2022 groups (median >20 h/week, IQR (0) in 2020 *vs.* 10-15 h/week, IQR (0) in 2019 and 5-10 h/week, IQR (1-5 h/week to 10-15 h/week) in 2022; p<0.01) (**Figure 4**). No significant difference in eating habits were evident among the groups.

## 4 Discussion

Our current data confirms the repeatedly reported, sharp increase in endocrinological consultations for suspected precocious or early puberty in girls, during the first waves of COVID-19 pandemic (8–21). As previously described, the increase in consultations was also reflected in an increase in CPP cases in 2020 compared to pre-pandemic values. Supporting the assumption of a different etiology between early and true precocious puberty, no difference in the number of cases of EP was observed throughout the four years.

The number of consultations for suspected precocious or early puberty in 2020 could have been affected by a selection bias due to the home confinement with elevated health anxiety that characterized the first phase of the pandemic. On the other hand, the significant increase of CPP cases in 2020 is supported by an objective diagnosis formulated by the same medical personnel among the different years.

For the first time, a gradual tendency towards a decrease of consultations and CPP cases during the evolution of the pandemic has been revealed, suggesting a downward trend of this phenomenon in concert with waning of the pandemic such that cases observed in 2022 were similar to the number of cases seen in 2019.

During 2021, the restrictive measures previously put in place to contain the pandemic were progressively relaxed and daily life activities returned to normal. Distant learning was gradually abandoned, at least in the primary schools, and children resumed face-to-face school activities. Group activities in leisure time and outdoor physical exercise resumed.

In a previous study (19), we correlated the increase of precocious puberty cases with home confinement, lack of physical exercise and the significant increase of daily screen time (both for studying and for leisure activities). These profound changes could have acted as stressors triggering the onset of puberty. The results of the present study seem to confirm the impact of lifestyle changes on pubertal timing.

Although there is no conclusive data on the association between poor physical activity and precocious puberty, a recent meta-analysis has confirmed that regular exercise training substantially increases adiponectin levels in obese children (27). Adiponectin, one of the most relevant adipokines secreted by mature adipocytes, has been demonstrated to suppress kisspeptin gene transcription and GnRH secretion by hypothalamic neurons, playing an inhibitory role in the onset of puberty (28, 29).

Beyond the physical benefits of exercise, several studies reported a positive association between physical activity and psychological well-being in children and adolescents. A sedentary lifestyle has been related to both depression and lower life satisfaction and happiness, while promoting physical activity and decreasing sedentary behavior might protect mental health (30). Early studies (31, 32) suggested that psychological stress itself (due to insecure bonds with parents or parental conflicts) might modify pubertal timing. A recent study reported that anxiety and other internalizing symptoms in pre-pubertal girls are associated with early pubertal onset, independently from maternal education anxiety, BMI, and ethnicity (33).

Several studies have recently investigated the effects of exposure to electromagnetic fields on melatonin (34–37). Exposure to electromagnetic fields has been associated with decreased melatonin production *in vitro*, as well as with a decreased pineal and plasma melatonin and its urinary metabolites (35). Nighttime serum melatonin levels are highest in infants and young children and decrease progressively by 80% throughout childhood and adolescence, nocturnal melatonin levels drop in parallel with sexual maturation (38, 39). Animal models have also shown that a reduction in melatonin may accelerate pubertal development (40) and that the administration of melatonin suppress GnRH secretion (41). A recent study performed on immature female rats differentially exposed to a light spectrum predominantly emitted by LED (light-emitting diode) screens, showed a faster pubertal maturation in rats bathed with the blue-tinged light for longer bouts (42). The combination of this data suggests that a greater use of electronic devices leads to a reduction in melatonin levels, which in turn triggers the endocrine changes culminating in the earlier onset of puberty (43).

Another study reported more frequent late bedtime, sleep disturbances, excessive somnolence, sleep breathing disorders and sleep-wake transition disorders in girls diagnosed with CPP during the Italian lockdown (15).

Published data analyzing the impact of overweight and obesity on the rise of CPP cases are conflicting (8, 13, 44, 45). Interestingly, we did not find any significant difference in BMI SDS at CPP diagnosis across the four years of observation, suggesting that overnutrition and overweight do not represent determining factors in this context.

All the mentioned factors (inactivity, increased screen time, sleep disturbances, and stress) may have contributed to the sharp increase in CPP cases, acting directly on the HPO axis. The retrospective design of the study does not allow identifying which factor predominates over the others. Indeed, the speed and reversibility of the phenomenon and the absence of differences in the anthropometric characteristics of the groups (in particular, BMI unchanged over the years) allows us to rule out already known risk factors for CPP (such as endocrine disruptors, obesity, or epigenetic factors). In support of this hypothesis, we observed lower basal LH levels and the less evident BA advancement in the 2020 CPP cases, compared to 2019. This could suggest that life-style changes can only act as weaker triggers of GnRH secretion with a transient effect on pubertal timing.

A single study from Korea described an almost doubled CPP incidence in 2021, in comparison with 2016, with a concurrent increase in the proportion of boys (19.55% *vs.* 9.21%) (11). As in the majority of the published studies, we reported an increase of CPP cases uniquely in girls. This fact seems to confirm that male CPP, in its rarity, is mostly related to organic disorders and/or genetic factors and less influenced by environmental changes.

We are aware that the major limitation of this study is its retrospective design, which did not allow us to obtain more data on factors potentially influencing GnRH secretion, but to our knowledge, this is the first study that describes a progressive downward trend in CPP cases during the post-pandemic period in 2022 to near pre-pandemic levels.

In conclusion, the sharp increase of CPP cases in girls during the first pandemic wave in mid-2020 seems to give way to a gradual downward trend, concurrently with the easing of the restrictive measures, returning to the pre-pandemic incidence of CPP in 2022. This suggests that the drastic lifestyle changes, as lack of physical exercise, increased screen time, sleep disturbances, and stress, may represent weak and reversible triggers on the central “biological clock” controlling timing and tempo of puberty.

## Data availability statement

The raw data supporting the conclusions of this article will be made available by the authors, without undue reservation.

## Ethics statement

The studies involving human participants were reviewed and approved by Bambino Gesù Children’s Hospital. Written informed consent from the participants’ legal guardian/next of kin was not required to participate in this study in accordance with the national legislation and the institutional requirements.

## Author contributions

CB and MCa conceptualized and designed the study. GB, LC, LP, TT, and MCh collected data. LC and MCh performed statistical analysis. CB, LC, and MCh drafted the initial manuscript, and reviewed the manuscript. MCa and CB revised the manuscript. All authors contributed to the article and approved the submitted version.
